# Role of the general practitioner in the care of *BRCA1* and *BRCA2* mutation carriers: General practitioner and patient perspectives

**DOI:** 10.1002/mgg3.464

**Published:** 2018-10-11

**Authors:** Pierre Vande Perre, Daniel Toledano, Carole Corsini, Elsa Escriba, Marine Laporte, Helena Bertet, Kevin Yauy, Alain Toledano, Virginie Galibert, Karen Baudry, Lucie Clotet, Elodie Million, Marie‐Christine Picot, David Geneviève, Pascal Pujol

**Affiliations:** ^1^ Department of Cancer Genetics Montpellier University Hospital (CHRU) Montpellier France; ^2^ Université Toulouse III Paul Sabatier Toulouse France; ^3^ Department of Cancer Genetics Breast Disease Center Saint Louis Hospital Paris France; ^4^ Epidemiology and Clinical Research Department INSERM U1411 Clinical Investigation Centre Montpellier University Hospital Montpellier France; ^5^ Department of Genetics Montpellier University Hospital (CHRU) Montpellier France; ^6^ University of Montpellier Montpellier France; ^7^ Department of Radiotherapy Hartmann Radiotherapy Center American Hospital of Paris Neuilly France; ^8^ University Department of General Medicine University of Montpellier Montpellier France; ^9^ Department of Medical Statistics INSERM U1046 CNRS UMR 9214 University of Montpellier Montpellier France

**Keywords:** *BRCA1*, *BRCA2*, general practitioners, genetic screening, hereditary breast and ovarian cancer syndrome

## Abstract

**Background:**

General practitioners (GPs) have an increasing role in referring patients with putative mutation in *BRCA1/2* genes for genetics consultation and for long‐term follow‐up of mutation carriers.

**Methods:**

We compared the expectations of the GPs’ role according to *BRCA1/2* mutation carriers and to GPs themselves.

**Results:**

Overall, 38% (58/152) of eligible GPs and 70% (176/252) of eligible patients were surveyed. Although 81% of GPs collected the family history, only 24% considered that they know criteria indicating genetics consultation and 39% sufficient knowledge of *BRCA1/2* guidelines to answer patients’ questions. Twelve% of GPs were aware of the French national guidelines. Among unsatisfied patients, 40% felt that their GP was able to answer (moderately, sufficiently, or completely) specific questions about BRCA1/2 care as compared with 81% in satisfied patients. Only 33% of GPs reported being informed directly by the geneticist about the patients’ results. GPs’ main expectations for their role in BRCA1/2 carrier care were psychological support and informing relatives about screening (72% and 71%, respectively), which contrasts with the perceptions of patients, who mainly requested medical advice for *BRCA1/2*‐related care (51%).

**Conclusion:**

There is an important need for GP training and enhancing interactions between GPs and geneticists to improve the GP's role in *BRCA1/2* screening and management.

## 1 INTRODUCTION

Deleterious mutations of *BRCA1* and *BRCA2*, the two major genes involved in hereditary breast and ovarian cancer syndrome (Cleton‐Jansen et al., 1995; Goldgar et al., 1994), are found in 1 per 300–500 women in the Caucasian general population (Anglian Breast Cancer (ABC) Study Group, 2000; Peto et al., 1999). The high risk of breast and ovarian cancer (Antoniou et al., 2003; Chen & Parmigiani, 2007; Giannakeas & Narod, 2018) in this population requires knowledge of personal and familial criteria for genetic counseling as well as specific long‐term follow‐up for screening and prevention strategies (Daly et al., 2017).

The expansion of genetic testing indications and the use of targeted therapies (such as PARP inhibitors) (Konecny & Kristeleit, 2016), in addition to conventional screening, has increased the number of individuals identified as carrying *BRCA1/2* mutations. Hence, the role of general practitioners (GPs) in identifying an indication for genetic testing and long‐term follow‐up of patients is crucial. Still, the involvement of GPs in the *BRCA1/2* care process remains mostly unexplored.

The aim of this study was to evaluate the role of GPs in the care of *BRCA1/2* mutation carriers and to compare the expectations of *BRCA1/2* mutation carriers for the GP role with those of GPs themselves.

## 2 MATERIAL AND METHODS

### 2.1 Design of the study

Our study was a multicentric, descriptive, prospective, clinical, double unpaired cohort study performed from July to September 2017. It was a descriptive cross‐sectional observational, noninterventional research. The study protocol was reported by the Clinical Investigation Center of University Hospital of Montpellier to ClinicalTrials.gov (Identifier: NCT03211611) on July 7, 2017.

### 2.2 Ethics

This study involved the collection of epidemiological, clinical, and genetic data from the medical files of patients and survey completion by email, telephone/fax, or mail. There was no medical intervention or change in patient health care. The study's protocol was approved by the ethics committee of Montpellier University Hospital and was declared to the National Commission for Computing and Liberties (CNIL, Supporting Information Appendix S2). All patients gave their written informed consent to participate. Anonymity was guaranteed for both GPs and patients by randomly assigning an anonymous identification number for collection of survey data.

### 2.3 Patient enrollment and assessment

**Figure 1:**
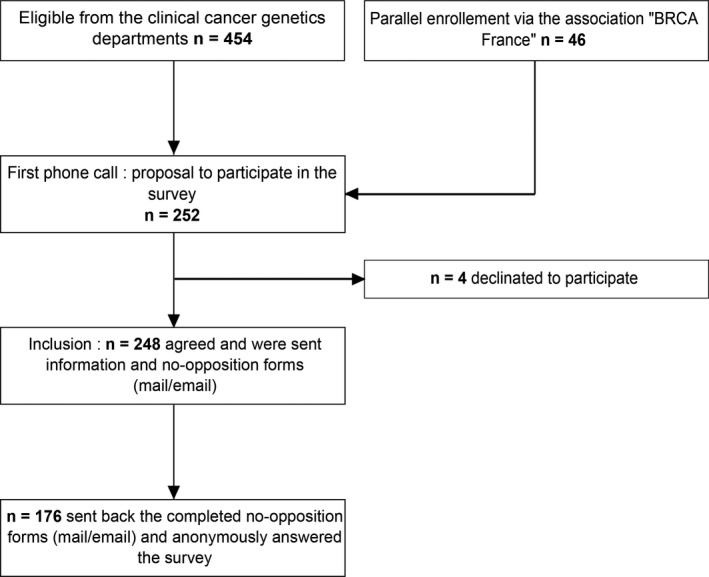
Flow of patients in the study

The flow of patients is in Figure 1. The recruitment of *BRCA1/2* mutated carriers was multicentric, involving cancer genetics units attached to the University Hospital of Montpellier (Centre Hospitalier Universitaire, CHU; Arnaud de Villeneuve Hospital, Montpellier Cancer Institute, Béziers Hospital, Perpignan Hospital), the Hartmann Oncology Center, and the Institut Hospitalier Franco‐Britannique in Levallois‐Perret. A subpopulation of *BRCA1/2* mutation carriers from the patient association BRCA France was enrolled via the association “BRCA France” on a voluntary basis.

We included women older than 18 years with genetically identified *BRCA1/2* germline mutation, with or without a cancer history, who were registered under the national social security scheme. Patients under guardianship or trusteeship, who were unable to consent for research or those protected by law, were not included. Patients were called by phone to inform them about the study's purpose and confidentiality of their responses. They were informed that the cancer genetics department of the CHU of Montpellier managed the study and that the BRCA France patient association was participating in patient recruitment.

An information and consent statement was sent by mail or email (Supporting Information Appendix S1). Once they signed and returned the consent form, the included patients completed a questionnaire containing 17 questions (closed dichotomous questions, multiple‐choice closed questions, conditional question, and questions with rating scales). This survey was organized in a multistep process, exploring demographic characteristics (age, distance from their living place to the GP's office or the cancer genetics center) and assessing medical management before the initial cancer genetics consultation and while awaiting the results. Then, the announcement of the results and the preventive treatment choices were explored. Finally, the medical follow‐up and the patient's satisfaction with the involvement of their GP in their specific follow‐up care related to *BRCA1/2* mutation carriage (primary endpoint) were assessed. This questionnaire is provided in Supporting Information Appendix S3. Patients could use their anonymous identification number to complete this questionnaire by phone, mail, or email (using a Google Form questionnaire). In the absence of an answer, patients were phoned or emailed twice.

The primary endpoint for patient assessment was rate of patients satisfied with the role their GP plays in their care related to *BRCA1/2* mutation carriage. Secondary endpoints were the patient's assessment of the GP's most important role in *BRCA1/2*‐related care and patients’ expectations of the GP's role.

### 2.4 GP enrollment and assessment

**Figure 2:**
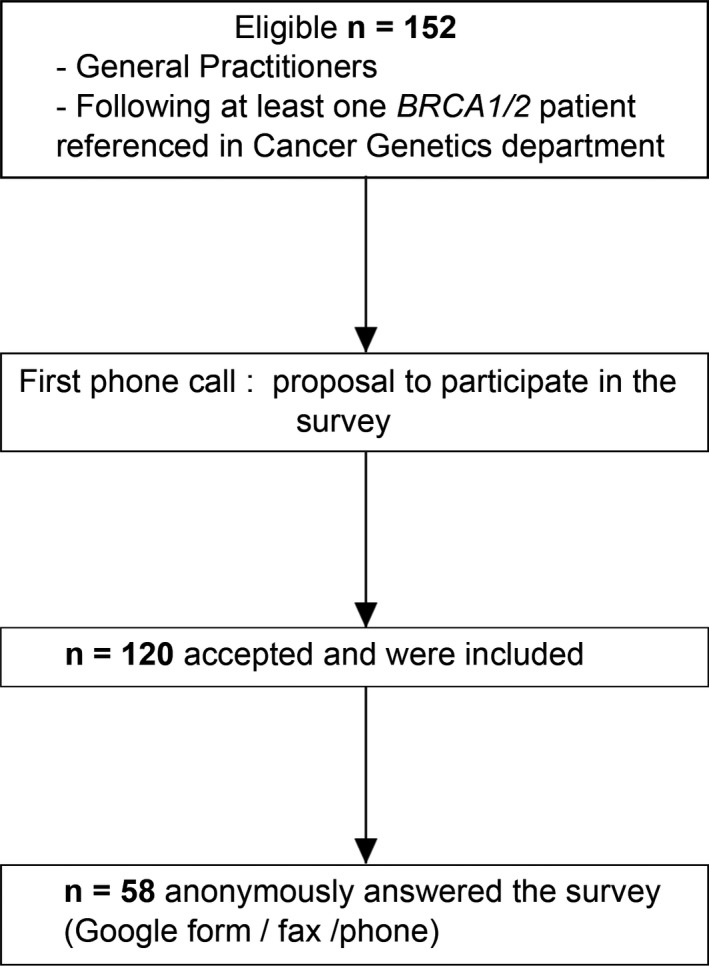
Flow of general practitioners (GPs) in the study

The flow of GPs in the study is in Figure 2. The Medifirst and DxCare secure medical software used in routine practice in the cancer genetics department of Montpellier University Hospital lists patients carrying mutations in *BRCA1* or *BRCA2* and identifies their referenced GPs, respectively. GPs were contacted by phone to explain the study and the method of anonymization. We included GPs who agreed to participate during this first telephone contact. We reminded them of their patient's name when they asked for it.

General practitioners self‐assessment of *BRCA1/2* carriers’ care was examined by a survey with 19 closed questions (Supporting Information Appendix S4). The survey items covered the demographic characteristics of GPs (sex, age, and place of practice) and a self‐assessment of the GPs’ knowledge of *BRCA1/2* mutations predisposing to cancer and their practices. This questionnaire was anonymized with the anonymous identification number previously communicated by email or by fax to GPs. GPs could answer the questionnaire by using Google Form or during a phone call or send the completed form by fax. In the absence of an answer, GPs were phoned or emailed twice. Any questionnaire even incomplete was considered.

The primary endpoint of the GP assessment was the rate of GPs considering that they had enough knowledge for appropriate care of patients with *BRCA1/2* mutation. This self‐assessment was characterized by three items:

- The self‐estimated level of systematic research for family history of cancer from the history taking with patients,
- The self‐perceived knowledge of the criteria required to refer the patient for cancer genetics consultation,
- The self‐evaluated ability to answer questions from patients about *BRCA1/2* mutations.

Secondary endpoints were the rate of GPs seeking cancer genetics training and that of GPs being informed about a medical report from the oncogeneticist about their patient's *BRCA1/2* mutation status.

### 2.5 Statistical analysis

Data entry and statistical analysis involved using Excel and BiostaTGV (Institut Pierre Louis d'Epidémiologie et de Santé Publique UMR S1136, https://biostatgv.sentiweb.fr). Data are presented as number (%; range) or mean ± *SD*. Results were analyzed by sex for GPs and the satisfaction criterion for patients by using the chi‐square or Fisher test. *p* < 0.05 was considered statistically significant.

## 3 RESULTS

### 3.1 Patient assessment

Overall, 424 patients from the Montpellier University Hospital oncogenetics department and 30 from the Hartmann Oncology Center were eligible; 46 additional patients were enrolled voluntarily via the BRCA France website. Among the 500 patients, 252 (50.4%) were telephoned or emailed: 16 (6.3%) agreed to answer by phone, 28 (11.1%) by mail, and 204 (81%) by email. Only four patients (1.6%) refused to participate, including one who was her own GP. Finally, from July to September 2017, 176/252 (69.8%) questionnaires were returned: 16 patients answered by telephone, 15 by mail, and 145 by email. For the 176 respondents, the mean age was 49.1 ± 13.68 years (range: 22–85); (Supporting Information Appendix Table S5); 101/175 (57.7%) had a personal history of cancer related to *BRCA1/2*. Most lived within 10 km of their GP's office (137/169, 81%), but the distance to the genetics department was more variable. A total of 46 (26.4%), 57 (32.8%), and 46 (26.4%) patients lived in urban, semirural, and rural areas, respectively.

Overall, 132/175 (75.4%) women were satisfied with their GP's role in their care (Table 1 and Supporting Information). Geographical distance to the GP office and the cancer genetic center did not affect the level of patient satisfaction (no difference in distance between satisfied and unsatisfied patients’ subgroups).

**Table 1:** Characteristics of patients with *BRCA1/2* mutation by their satisfaction or not with the involvement of their GP in their specific follow‐up care (*n* = 176)

Characteristics	Answers	Unsatisfied *n* = 43(%)	Satisfied *n* = 132 (%)	Total *N*	*p* [Link]
Personal history of cancer	Yes	24 (55.8)	77 (58.3)	101	0.77
	No	19 (44.2)	55 (41.7)	74	
GP consultation before geneticist	Yes	3 (7)	43 (32.5)	46	0.0009
	No	40 (93)	89 (67.5)	129	
Ability of GP to answer *BRCA1/2‐*specific questions from patients	1‐Not at all	23 (60.5)	22 (18.2)	45	0.0001
	2‐A little				
	3‐Moderately	11 (29)	40 (33)	51	
	4‐Sufficiently	4 (10.5)	59 (48.8)	63	
	5‐Completely				
Personal research	Yes	29 (67.4)	52 (39.7)	81	0.0016
	No	14 (32.5)	79 (60.3)	93	
GPs should provide psychological support before disclosure of the results	1‐Totally disagree	35 (81.4)	95 (72)	130	0.26
	2‐Somewhat disagree				
	3‐Neither agree nor disagree	4 (9.3)	19 (14.4)	23	
	4‐Rather agree	4 (9.3)	18 (13.6)	22	
	5‐Totally agree				
Possibility to ask the geneticist about *BRCA1/2* care and follow‐up questions	Yes	33 (76.7)	114 (86.4)	147	0.09
	No	9 (21)	16 (12.1)	25	
Practitioner responsible for strategy option (breast screening or surgery)	Oncogeneticist	35 (94.6)	74 (66.7)	109	0.0032
	GP	2(5.4)	37 (33.3)	39	
Practitioner responsible for breast surveillance	GP	1 (2.3)	32 (24.4)	33	0.0016
	Gynecologist	41 (97.7)	99 (75.6)	140	
	Oncologist				
	Oncogeneticist				
	Radiologist				
	No one				

*Notes*

Any questionnaire answered, even incomplete was considered.

GP: general practitioner.

*p* < 0.05 by chi‐square or Fisher exact test.

Before the genetics results disclosure, only 46/175 (26.3%) patients consulted their GP. Consultation with the GP before the genetics consultation was more frequent for satisfied than unsatisfied patients (32.5% vs. 7%, *p* < 0.001). Satisfaction of patients was associated with the reported clinical breast surveillance role performed by GPs (19% 33/173, Table 1; *p* < 0.01). The ability of GPs to answer questions was deemed significantly higher for satisfied than unsatisfied patients (*p* < 0.01). Additional information was searched more often independently by unsatisfied than satisfied patients (*p* < 0.01).

Only, 22/175 (12.5%) patients considered the psychological support of GPs necessary pending the genetics results.

### 3.2 GP assessment

From July to September 2017, 152 GPs were called and 120 agreed to participate and were included in the study; 58 (38.2%) answered the questionnaire.

The GPs were mostly older than 50 years (46/58, 79.3%) and 24/58 (41%) were women. A total of 30 (52%), 21 (36%), and 7 (12%) practiced in urban, semirural, and rural areas, respectively (Supporting Information Appendix Table S6). Overall, 47/58 (81%) GPs collected the family history and only 14/58 (24%) considered they had sufficient knowledge of the indication criteria for genetics consultation (Table 2). Also, 28/46 (69.7%) considered that they were not able to answer patients’ questions about *BRCA1/2* guidelines. Many GPs (24/58, 72.4%) felt not included (24/58) or that they had a minor role (18/58) in the care of their patients. GPs’ answers are detailed in supplementary material.

**Table 2:** Rate of knowledge of GPs for appropriate care of patients with *BRCA1/2* mutation by sex (*n* = 58)

Characteristics	Total	Answer (*N*)	Women *n* = 24 *N* (%)	Men *n* = 24 *N* (%)	*p* [Link]
Family history of cancer systematically referred by GP	58	Yes (47)	22 (91.7)	25 (73.5)	0.08
		No (11)	2 (8.3)	9 (26.5)	
Knowledge of referral guidelines in cancer genetics consultation	58	Yes (14)	9 (37.5)	5 (14.7)	0.04
		No (44)	15 (62.5)	29 (85.3)	
Ability of GP to answer *BRCA1/2* management and follow‐up questions	46	Yes (18)	10 (58.8)	8 (27.6)	0.03
		No (28)	7 (41.2)	21 (72.4)	

*p* < 0.05 by chi‐square test.

After stratification by sex, female GPs were significantly better acquainted with the criteria for referring patients to cancer genetics departments (*p* = 0.04) and felt more competent to answer patients’ questions (*p* = 0.03) than male GPs (Table 2).

A total of 14/51 (27.5%) GPs were trained during their initial training to care for patients with a *BRCA1/2* mutation (Supporting Information Appendix Table S6), in light of most responding GPs being older than 50 years (79.3%). Only 11.8% of the GPs attributed their knowledge on the subject to the referral guidelines of the French national cancer institute (INCa).

Overall, only 19/58 (32.8%) GPs reported receiving a letter from the geneticist.

### 3.3 Comparison between GP and patient responses

**Figure 3:**
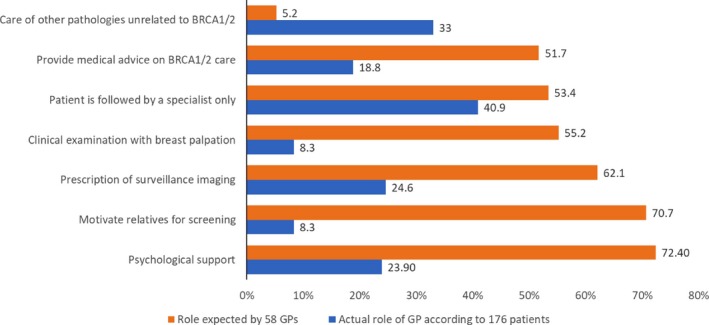
Comparison between the actual role of GPs in the BRCA1/2‐related care according to the patients (*n* = 176, in blue) and the GPs (*n* = 58, in orange)

**Figure 4:**
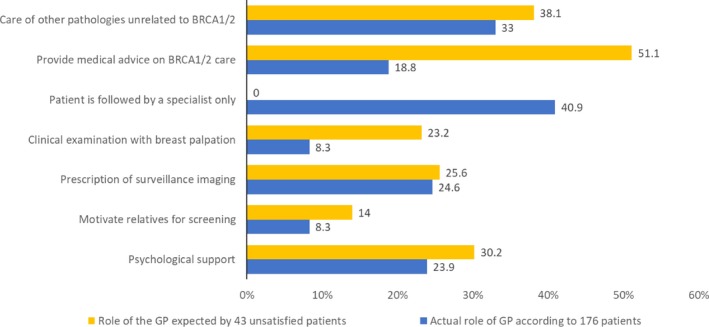
Comparison between the role of GPs according to the overall population of patients (*n* = 176 patients, in blue) and the unsatisfied patients subgroup (*n* = 43, in yellow)

We compared the overall results for several items of the questionnaires between the study groups. Figure 3 compares the actual role of GPs according to the 176 patients and what the 58 GPs expected as their future role in care of patients with *BRCA1/2* mutations: Patients mainly expected care of other pathologies other than *BRAC1/2* mutations from their GPs, and GPs mainly expected they would motivate relatives for screening and provide psychological support. Figure 4 compares the role of the GP according to the 176 patients with the role of the GP expected by the 43 unsatisfied patients: All patients expected they would be followed by the specialist and unsatisfied patients mainly expected advice about their medical *BRCA*‐related care from their GP.

## 4 DISCUSSION

We report a double cohort study that evaluated and compared the perceptions of patients and GPs concerning GPs’ involvement in care of patients with *BRCA1/2* mutation. This study revealed that GPs are poorly informed (both by specialists and by patients) of the result of the genetic *BRCA1/2* analysis: In our study, only 32.8% of GPs thought that they were notified of the genetics results for their patient. Therefore, it is not surprising that many GPs (72.4%) felt little involved in the *BRCA1/2* care of their patients. The inclusion of GPs in the *BRCA1/2* care process should be improved. Although a center effect could not be ruled out, we believe that a better information and return of the results of the genetics analysis to the GP are required.

Potential bias of this study is the representativity of the subset (38%) of the GPs answering the survey. However, it is likely that GPs responding may be interested by the question of *BRCA1/2* care and that the insufficiency of knowledges of guidelines may be underestimated.

Most GPs considered that they were not familiar with the criteria for referring patients to cancer genetics consultations (75.9%), which in a previous study was evaluated as one of the main elements of GPs’ cancer genetics training (Houwink et al., 2012). Only 17.2% of our GPs referred their patient with *BRCA1/2* mutation to the oncogenetics consultation, but most GPs looked for a family history of cancer during the consultation (81%).

The increased involvement of women GPs in referring patients with extreme risk of breast cancer and/or ovarian cancer to oncogenetics consultation was previously observed (Campbell et al., 2003). In addition, a European study showed that female GPs had a more positive attitude about prophylactic mastectomy than male GPs and that knowledge of breast/ovarian cancer genetics was associated with a more positive attitude about prophylactic mastectomy (Den Heijer et al., 2013). Therefore, our sample of GPs seems representative of others described in the literature.

Our GPs’ self‐assessment was not controlled by a test of their effective knowledge. The absence of knowledge of the referral guidelines of the INCa is consistent with their self‐reported lack of awareness about indications of genetic tests and medical guidelines on *BRCA1/2*. A European study of cancer risk assessment, predictive testing, and management by GPs and breast surgeons reported that physicians of all evaluated countries tend to fail to take into account the paternal side of the family when collecting the family history (Nippert et al., 2014).

In France, recommendations for breast cancer screening in the general population with estimated “moderate risk” by mammography every 2 years between age 50 and 74 years frequently involve GPs. Therefore, GPs need the necessary knowledge to distinguish breast cancer risk levels and refer the patient to genetics counseling when appropriate.

General practitioners are poorly trained in the management of BRCA1/2‐associated cancer risk, both in terms of initial training and continuing education. Only one‐third of the GPs answered our questionnaire (38.2%). Most GPs assessed were older than 50 years, which is consistent with data from the atlas of French medical demography from the “Ordre des médecins” and may explain the lack of initial training on *BRCA1/2* management. The INCa guidelines were considered the main source of knowledge for only 11.8% of GPs. In a 2013 European survey, only 30% of French GPs considered prophylactic mastectomy an option for an unaffected female *BRCA1/2* mutation carrier (as compared with 27%, 85%, and 92% in Germany, the Netherlands, and United Kingdom, respectively) (Den Heijer et al., 2013).

There is a real need for training GPs in the care of such patients, and most are interested in training (79.6%), which agrees with previous European (Nippert et al., 2014), US (Friedman, Cooper, Webb, Weinberg, & Plon, 2003; Friedman, Plon, Cooper, & Weinberg, 1997), Australian (Teng & Spigelman, 2014), and South African (Van Wyk, Wessels, Kromberg, & Krause, 2016) studies.

Most GPs (69.7%) consider that they do not have the necessary knowledge to answer their patient's questions. Therefore, their main expected role in caring for these patients is limited to psychological support (72.4%) and to motivate relatives to undergo screening (70.7%). However, this finding does not agree with patients who want their GPs to be involved in their surveillance protocol and their care: Most unsatisfied patients (51.1%) gave priority to advice regarding their care breast/ovarian care. Overall, 12.5% of patients considered the psychological support of GPs necessary pending the results and, after diagnosis, 23.9% considered that the psychological support was one of the GP's roles. This request of patients to see GPs involved in cancer genetics care is consistent with a previous similar study (Miller et al., 2010).

Therefore, the expectation of patients carrying a *BRCA1/2* mutation must be met by including GPs more efficiently in the *BRCA1/2* care process and providing specific training to GPs. Previous studies have shown the effectiveness and ease of establishing training in oncogenetics (Houwink et al., 2014; Scheuner et al., 2014; Watson et al., 2001) or rare genetic diseases (Carroll et al., 2009; Paneque et al., 2016) for GPs.

With the increasing number of *BRCA1/2* mutation carriers identified in the general population and the need for a long‐term follow‐up, GPs will have an increasing role in the care of such patients. This work points to the crucial need to improve GPs’ medical training in the indication for tests and *BRCA1/2* care guidelines. It also reports a major expectation of patient mutation carriers for a higher involvement of GPs in their medical care.

## CONFLICT OF INTEREST

The authors declare that they have no competing interests. The authors declare that this work was not funded by industrial or commercial companies.
