# Supplementary material for: Role of the general practitioner in the care of BRCA1 and BRCA2 mutation carriers: General practitioner and patient perspectives
Source: Mol Genet Genomic Med. 2018 Oct 11;6(6):957–65. doi: 10.1002/mgg3.464 (PMC6305637; doi:10.1002/mgg3.464)
Supplement: Supplementary file 1 [file MGG3-6-957-s001.docx]

Document 1: Information and no-opposition letter sent to patients.

"Evaluation of the current role of the general practitioner in the care of patients with BRCA gene mutation"

Madame,

We invite you to participate in the research protocol entitled « Evaluation of the current role of the general practitioner in the management of patients with BRCA gene mutation » coordinated by the Professor Pascal PUJOL in the cancer genetics department of the Montpellier University Hospital.

We invite you to read carefully this information note which aims to answer any questions you may have before making your decision to participate or not in this research.

As part of this research, we focus on female mutation carriers of *BRCA1/2* to assess the current role of the general practitioner in their care management. The results of our research will allow in the future to adapt the place of the general practitioner in relation to the expectations and needs of patients carrying this type of mutations.

As part of this descriptive, multicenter, prospective cohort genetic epidemiological research, we wish to collect from you, via your medical file and from the doctor who follows you, medical and clinical data. Indeed, if you agree to participate in this research, we will ask you to complete (phone, mail or email) a short questionnaire (duration about 10 min) consisting of closed questions dichotomous issues closed multiple choice, a question conditional, questions with rating scales. In addition, we will ask the doctor who follows you to answer a questionnaire consisting of closed questions, closed multiple-choice questions, and questions with an evaluation scale. These questionnaires will allow us to answer the objective of our research.

This research is not intended to change your medical management. There will be no additional consultation or examination to those essential to the follow-up of your pathology, nor of modifications of the treatment prescribed by your doctor. Only data entered in your medical file will be collected as well as data from the questionnaire.

You have a right of opposition, without consequences on the continuation of the treatment or the quality of the care which will be provided to you. In addition, your participation in this research is voluntary and you can interrupt at any time without justification.

No immediate individual benefit is expected for research participants. Nevertheless, this research would make it possible to adapt the general practitioner's position to the expectations and needs of patients with mutations in the *BRCA1/2* gene.

**This research received a favorable opinion from an ethics committee.**

This study is in accordance with the Data Protection Act to the 003 reference methodology (MR003) that frames treatments including health data made in the context of research.

**Data Collection and Privacy**

If you do not object to this research, the data collected will be anonymized. The collection will be performed by health personnel bound by professional secrecy and under the responsibility of Professor Pascal PUJOL. The recipients of the data necessary for the purpose of this research are Professor Pascal PUJOL (Coordinator), ESCRIBA Elsa, general practitioner resident, LAPORTE Marine, general practitioner resident.

As part of this research, an automated and anonymized personal data processing will be implemented for the analysis of search results. Your data will be identified by a code number (Chronological entry number in the search) guaranteeing your complete anonymity and they will be kept for a period of 12 months (duration of research) within the oncogenetics department of Montpellier University Hospital. f you wish, the overall results of this work will be communicated to you at its conclusion by the doctor in charge of your follow-up. No data will allow your identification in scientific reports or publications resulting from this research.

For any information concerning this research or to express your right of access, rectification or opposition, you can contact by mail / mail:

ESCRIBA Elsa & LAPORTE Marine;

E-mail: these2017brca@gmail.com

Adresse: service oncogénétique, CHU ARNAUD DE VILLENEUVE, 371 Av. du Doyen Gaston Giraud, 34090 Montpellier.

Be assured that your participation is extremely valuable to us. We thank you in advance for the help you provide to this research.
