# Supplementary material for: Role of the general practitioner in the care of BRCA1 and BRCA2 mutation carriers: General practitioner and patient perspectives
Source: Mol Genet Genomic Med. 2018 Oct 11;6(6):957–65. doi: 10.1002/mgg3.464 (PMC6305637; doi:10.1002/mgg3.464)
Supplement: Supplementary file 2 [file MGG3-6-957-s002.pdf]

Document 2: declaration to the National Commission for Computing and Liberties (CNIL), provided in French.

CNIL.
[illegible] PARIS cedex 02
T. 01 53 73 22 22 F. 01 53 73 22 00
www.cnil.fr

RÉCÉPISSÉ

DÉCLARATION DE CONFORMITÉ À UNE MÉTHODOLOGIE DE RÉFÉRENCE

Numéro de déclaration

1984895 v 0

du 25 août 2016

CENTRE HOSPITALIER UNIVERSITAIRE DE MONTPELLIER
DIRECTION GENERALE
191 AVENUE DU DOYEN GASTON GIRAUD
CENTRE ADMINISTRATIF A. BENECH
34295 MONTPELLIER CEDEX 5

A LIRE IMPERATIVEMENT

La délivrance de ce récépissé atteste que vous avez transmis à la CNIL un dossier de déclaration formellement complet. Vous pouvez désormais mettre en oeuvre votre traitement de données à caractère personnel.

La CNIL peut à tout moment vérifier, par courrier, par la voie d'un contrôle sur place ou en ligne, que ce traitement respecte l'ensemble des dispositions de la loi du 6 janvier 1978 modifiée en 2004. Afin d'être conforme à la loi, vous êtes tenu de respecter tout au long de votre traitement les obligations prévues et notamment :

1) La définition et le respect de la finalité du traitement,
2) La pertinence des données traitées,
3) La conservation pendant une durée limitée des données,
4) La sécurité et la confidentialité des données,
5) Le respect des droits des intéressés : information sur leur droit d'accès, de rectification et d'opposition.

Pour plus de détails sur les obligations prévues par la loi « informatique et libertés », consultez le site internet de la CNIL : www.cnil.fr.

Organisme déclarant

Nom : CENTRE HOSPITALIER UNIVERSITAIRE DE MONTPELLIER

Service : DIRECTION GENERALE

Adresse : 191 AVENUE DU DOYEN GASTON GIRAUD CENTRE ADMINISTRATIF A. BENECH

Code postal : 34295
Ville : MONTPELLIER CEDEX 5

N° SIREN ou SIRET :
263400160 00382

Code NAF ou APE :
8610Z

Tél. : 0437339458
Fax. : 467336773

Traitement déclaré

Finalité : MR3 - Recherches dans le domaine de la santé sans recueil du consentement

Transferts d'informations hors de l'Union européenne : Non

Fait à Paris, le 25 août 2016
Par délégation de la commission

Isabelle FALQUE PIERROTIN
Présidente
