# Supplementary material for: Role of the general practitioner in the care of BRCA1 and BRCA2 mutation carriers: General practitioner and patient perspectives
Source: Mol Genet Genomic Med. 2018 Oct 11;6(6):957–65. doi: 10.1002/mgg3.464 (PMC6305637; doi:10.1002/mgg3.464)
Supplement: Supplementary file 3 [file MGG3-6-957-s003.docx]

Document 3: Questionnaire (17 questions) for patient carriers of *BRCA1/2* mutations.

PATIENT QUESTIONNAIRE

**Anonymity number**:……………………………………..

1. **Age:**

2. **Residence:** □ urban; □ semi-rural; □ rural

3. **Distance from the place of life**

**- to the center of cancer genetics** <10km / 10 to 50 km / 50 to 100km / >100km;

- **to the** **GP office**: < 10 km / 10 to 30 km/ >30 km

4. **Personal history of cancer related to the *BRCA1/2* mutation:** □ Yes □ No

BEFORE ONCOGENETIC CONSULTATION

5. **Did you consult your GP?** □ Yes □ No

6. **If so, why**?

□ Need information about the consultation

□ Need a letter for the geneticist

□ Other..............................................................

7. **Did you feel your GP able to fulfill your *BRCA1/2* specific questions?**

1 Not at all; 2 A little; 3 Moderately, 4 Sufficiently, 5 Completely

8. **Did you make personal research on the subject, on the internet for example?** (only one answer)

□ Yes, because you always do it.

□ Yes, because the answers from your GP were not enough.

□ No, because you were afraid of sad or false information.

□ No, you preferred to wait for the specialized consultation.

**WAITING FOR GENETIC TESTS RESULTS**

9. **Do you agree or disagree with this statement: You needed the help of your treating physician on a psychological level while waiting for the results.**

1 Totally disagree, 2 Somewhat disagree, 3 Neither agree nor disagree, 4 Rather agree, 5 Totally agree

**IN CANCER GENETICS CONSULTATION: DISCLOSURE OF THE RESULTS**

10. **Did you feel able to ask the geneticist all your questions?** (Only 1 answer)

□ Yes

□ Yes, but others came later, I was able to talk about it again with the geneticist

□ Yes, but others came later, I was able to talk about it again with the GP

□ No, I was shocked

□ No, I did not dare

13. **Who give you advice regarding breast strategy option (breast imaging or surgery)?**

□ The cancer geneticist

□ The GP

**ABOUT YOUR MEDICAL CARE**

14. **Which doctor coordinated your breast surveillance?**

□ GP: Yes/No

□ Gynecologist: Yes/No

□ Oncologist: Yes/No

□ Cancer geneticist: Yes/No

□ Radiologist: Yes/No

□ No one

15. **What is the role of your GP in your care?** (multiple choice possible)

□ Clinical screening with breast palpation

□ Prescription of surveillance imaging

□ Other pathologies only, unrelated to *BRCA1/2*

□ I am followed by specialists only

□ Medical advice concerning my care related to *BRCA1/2*

□ Psychological support

□ Explain the value of screening to relatives

□ Other: ….........................................................

16. **Are you satisfied with the role of your GP in your care?** □ Yes □ No

17**. If NO, what additional role (s) would you like him/her to take in your care**? (multiple choice possible)

□ Clinical screening with breast palpation

□ Prescription of surveillance imaging

□ Other pathologies only, unrelated to *BRCA1/2*

□ I am followed by specialists only

□ Medical advice concerning my care related to *BRCA1/2*

□ Psychological support

□ Explain the value of screening to relatives

□ Other: ….........................................................

18. **What role does your GP play that seems most important to you?** (Only 1 answer)

□ Clinical screening with breast palpation

□ Prescription of surveillance imaging

□ Other pathologies only, unrelated to *BRCA1/2*

□ I am followed by specialists only

□ Medical advice concerning my care related to *BRCA1/2*

□ Psychological support

□ Explain the value of screening to relatives

□ Other: ….........................................................
