# Supplementary material for: Role of the general practitioner in the care of BRCA1 and BRCA2 mutation carriers: General practitioner and patient perspectives
Source: Mol Genet Genomic Med. 2018 Oct 11;6(6):957–65. doi: 10.1002/mgg3.464 (PMC6305637; doi:10.1002/mgg3.464)
Supplement: Supplementary file 4 [file MGG3-6-957-s004.docx]

Document 4: General practitioner questionnaire (19 closed questions).

GENERAL PRACTITIONER QUESTIONNAIRE

**Anonymity number**:……………………………………..

1. **Age**: <40 years old / 40 to 50 years old / 50 to 60 years old / > 60 years old

2. **Gender**: □ Female □ Male

3. **Place of medical practice:** □ urban □ semi-rural □ rural

4**. Do you routinely look for a family history of cancer during history taking?** □ Yes □ No

5. **Did you discuss the topic of family predisposition with this patient?** □ Yes □ No

6. **Did you refer the patient to a cancer genetics consultation?** □ Yes □ No

7. **Do you know the criteria required to refer the patient to a consultation on cancer genetics?**

□ Yes □ No

8. **Did your patient come to see you for information before the cancer genetics consultation?**

□ Yes □ No

9. **Do you think you were able to answer questions from patient about *BRCA1/2* mutations?**

□ Yes □ No

10. **Were you sure about the information you gave her?** □ Yes □ No

11. **What was the main source of your knowledge?** (only 1 answer)

□ Initial formation

□ Continuing education

□ Literature: articles, books on the subject

□ Recommendations from the French National Cancer Institute (INCA)

□ The Internet

□ The cancer geneticist

12. **When your patient consulted with you, how long had you updated your knowledge?**

□ < 1 year □ 1 to 5 years □ 5 to 10 years □ > 10 years

13. **Did you do research on this topic after your patient consulted with you?** □ Yes □ No

14. **Would you be interested in specific training on cancer genetics as part of medical continuing medical education?** □ Yes □ No

15. **Once the mutation was identified in your patient, who informed you of the result?** (only 1 answer)

□ The patient

□ The gynecologist

□ The oncologist

□ The cancer geneticist

□ No one

16. **Did your patient came to ask for other information from you, after the disclosure of the results?** □ Yes □ No

17. **Did your patient ask you for advice about future care?**

□ No

□ About treatment

□ About screening

□ About prevention

18. **What role do you think you have played in the management of the *BRCA1/2* mutation?**

-1 None,

- 2 Minor

- 3 Moderate

- 4 Important

- 5 Highly important

19**. What role would you like to have later in your patient’s care?** (multiple answers possible)

□ Psychological support

□ Motivate relatives for screening

□ Medical imaging prescription

□ Clinical examination with breast palpation

□ I prefer that she be followed by a specialist

□ Medical advice on strategy options of care

□ Care of other pathologies unrelated to *BRCA1/2*
