# Supplementary material for: Role of the general practitioner in the care of BRCA1 and BRCA2 mutation carriers: General practitioner and patient perspectives
Source: Mol Genet Genomic Med. 2018 Oct 11;6(6):957–65. doi: 10.1002/mgg3.464 (PMC6305637; doi:10.1002/mgg3.464)
Supplement: Supplementary file 5 [file MGG3-6-957-s005.docx]

TableS1**:** Complete answers for patients (n=176).

| **Characteristics** | **Answers** | **Mean responses n (%)** | **Total (N)** |
| --- | --- | --- | --- |
| **Age, years (range), mean±SD** | 49.1 ± 13.68 [22 to 85] |  | 173 |
| **Residence** | Urban | 46 (26.4) | 174 |
|  | Semi-rural | 57 (32.8) |  |
|  | Rural | 46 (26.4) |  |
| **Distance to the center of cancer genetics, km** | <10 | 33 (19) | 174 |
|  | 10 to 50 | 65 (37.6) |  |
|  | 50 to 100 | 32 (18.4) |  |
|  | >100 | 44 (25.3) |  |
| **Distance to the** **GP office, km** | <10 | 137 (81) | 169 |
|  | 10 to 30 | 27 (16) |  |
|  | >30 | 5 (3) |  |
| **Personal history of cancer** | Yes | 101 (57.7) | 175 |
|  | No | 74 (42.3) |  |
| **GP consultation before geneticist** | Yes | 46 (26.3) | 175 |
|  | No | 129 (73.7) |  |
| **If so, why?** | Need information about the genetics consultation | 26 (56.5) | 46 |
|  | Need a letter for the geneticist | 12 (26.1) |  |
|  | Other | 8 (17.4) |  |
| **Did you feel your GP able to answer questions on the subject?** | 1-Not at all | 22 (13.8) | 159 |
|  | 2-A little | 23 (14.5) |  |
|  | 3-Moderatly | 51 (29) |  |
|  | 4-Sufficiently | 29 (18.2) |  |
|  | 5-Completely | 34 (21.4) |  |
| **Personal research on *BRCA1/2* predisposition?** (only one answer) | Yes, because you always do it. | 81 (46.6) | 174 |
|  | Yes, because the answers from your GP were not enough. |  |  |
|  | No, because you were afraid of sad or false information. | 93 (53.4) |  |
|  | No, you preferred to wait for the specialized consultation. |  |  |
| **GP’s psychological support before disclosure of the results** | 1-Totally disagree | 101 (57.7) | 175 |
|  | 2-Somewhat disagree | 29 (16.6) |  |
|  | 3-Neither agree nor disagree | 23 (13.1) |  |
|  | 4-Rather agree | 7 (4) |  |
|  | 5-Totally agree | 15 (8.6) |  |
| **Possibility to ask the geneticist *BRCA1/2* management and follow-up questions** (Only 1 answer) | Yes | 69 (40.1) | 172 |
|  | Yes, but others came later, I was able to talk about it again with the oncogeneticist | 61 (35.5) |  |
|  | Yes, but others came later, I was able to talk about it again with the GP | 17 (9.9) |  |
|  | No, I was shocked | 19 (11) |  |
|  | No, I did not dare | 6 (3.5) |  |
| **Practitioner responsible for strategy option (breast screening or surgery)** (multiple answers possible) | Oncogeneticist | 109 (73.7) | 148 |
|  | GP | 25 (16.9) |  |
|  | Both (GP and oncogeneticist) | 14 (9.5) |  |
| **Practitioner responsible for breast surveillance** (multiple answers possible) | GP | 33 (19) | 173 |
|  | Gynecologist | 86 (49.7) |  |
|  | Oncologist | 76 (43.9) |  |
|  | Oncogeneticist | 23 (13.3) |  |
|  | Radiologist | 75 (43.35) |  |
|  | No one | 0 |  |
| **Current role of the GP in patient** (multiple answers possible) | Clinical examination of screening with breast palpation | 13 (8.3) | 175 |
|  | Prescription of surveillance imaging | 43 (24.6) |  |
|  | Other pathologies only, unrelated to *BRCA1/2* | 58 (33) |  |
|  | I am followed by specialists only | 72 (40.9) |  |
|  | Medical advice concerning my care related to *BRCA1/2* | 33 (18.8) |  |
|  | Psychological support | 42 (23.9) |  |
|  | Explain the value of screening to relatives | 12 (8.3) |  |
|  | Other | 5 (2.8): 1 hormone therapy prescriber, 1 administrative role, 3 care coordinator |  |
| **Satisfaction regarding the role of the GP in patient care?** | Yes | 132 (75.4) | 175 |
|  | No | 43 (24.6) |  |
| **If NO, additional GP role(s) expected by patient** (multiple choice possible) | Clinical examination of screening with breast palpation | 10 (23.2) | 43 |
|  | Prescription of surveillance imaging | 11 (25.6) |  |
|  | Medical advice concerning my care related to *BRCA1/2* | 22 (51.1) |  |
|  | Psychological support | 13 (30.2) |  |
|  | Explain the value of screening to relatives | 6 (14) |  |
|  | Others | 4(9.3): 3 care coordinators, 1 listening role |  |
| **The most important GP current role** (Only 1 answer) | Clinical examination of screening with breast palpation | 11 (6.3) | 175 |
|  | Prescription of surveillance imaging | 21 (12.1) |  |
|  | Other pathologies only, unrelated to *BRCA1/2* | 67 (38.1) |  |
|  | I prefer to be followed by specialists only | 27 (15.4) |  |
|  | Medical advice concerning my care related to *BRCA1/2* | 38 (21.9) |  |
|  | Psychological support | 28 (16.1) |  |
|  | Explain the value of screening to relatives | 4 (2.3) |  |
|  | Others | 7 (3.9): 2 care coordinators, 2 comprehensive medical care, 2 relay with specialists, 1 proximity and availability |  |

GP: general practitioner
