# Supplementary material for: Role of the general practitioner in the care of BRCA1 and BRCA2 mutation carriers: General practitioner and patient perspectives
Source: Mol Genet Genomic Med. 2018 Oct 11;6(6):957–65. doi: 10.1002/mgg3.464 (PMC6305637; doi:10.1002/mgg3.464)
Supplement: Supplementary file 6 [file MGG3-6-957-s006.docx]

TableS2: Complete answers for GPs (n=58).

| **Characteristics** | **Answers** | **Mean responses n (%)** | **Total (N)** |
| --- | --- | --- | --- |
| **Age category, years** | <40 | 7 (12) | 58 |
|  | 40 to 50 | 5 (9) |  |
|  | 50 to 60 | 25 (43) |  |
|  | >60 | 21 (36) |  |
| **Sex** | Women | 24 (41) | 58 |
|  | Men | 34 (59) |  |
| **Place of medical practice** | Urban | 30 (52) | 58 |
|  | Semi-rural | 21 (36) |  |
|  | Rural | 7 (12) |  |
| **Discussion with patient about family predisposition** | Yes | 33 (56.9) | 58 |
|  | No | 25 (43.1) |  |
| **GP referred the patient to the cancer genetics consultation** | Yes | 10 (17.24) | 58 |
|  | No | 48 (82.76) |  |
| **GP consultation prior to geneticist** | Yes | 16 (28.07) | 57 |
|  | No | 41 (71.93) |  |
| **Certainty regarding the information given** | Yes | 10 (23.81) | 42 |
|  | No | 32 (76.9) |  |
| **Main source of GP knowledge** | Initial education | 14 (27.5) | 51 |
|  | Continuing education | 17 (33.3) |  |
|  | Literature: articles, books on the subject | 24 (47.1) |  |
|  | Recommendations from the French National Cancer Institute (INCA) | 6 (11.8) |  |
|  | Internet | 16 (31.4) |  |
|  | Cancer genetics specialist | 11 (21.6) |  |
| **Date of last update of knowledge, years** | < 1 | 10 (20) | 50 |
|  | 1 to 5 | 30 (60) |  |
|  | 5 to 10 | 4 (8) |  |
|  | >10 | 6 (12) |  |
| **Research on *BRCA1/2* after consultation** | Yes | 23 (43.4) | 53 |
|  | No | 30 (56.6) |  |
| **Interest in specific training on cancer genetics as part of medical continuing medical education (FMC)** | Yes | 43 (79.63) | 53 |
|  | No | 11 (20.37) |  |
| **Source of patient results** | Patient | 24 (41.4) | 58 |
|  | Oncologist | 21 (36.2) |  |
|  | Cancer geneticist | 19 (32.8) |  |
|  | Gynecologist | 2 (3.4) |  |
|  | No one | 4 (6.9) |  |
| **Patient request for information after results disclosure** | Yes | 18 (31.58) | 57 |
|  | No | 39 (68.42) |  |
| **Patient request for advice about future care** | No | 34 (61.8) | 55 |
|  | About treatment | 13 (23.6) |  |
|  | About screening | 10 (18.2) |  |
|  | About prevention | 10 (18.2) |  |
| **Perceived role of GP in the care of the patient** | 1-None | 24 (41.38) | 58 |
|  | 2-Minor | 18 (31.03) |  |
|  | 3-Moderate | 14 (24.14) |  |
|  | 4-Important | 2 (3.5) |  |
|  | 5-Highly important | 0 |  |
| **GP expected role in patient care** (multiple answers possible) | Psychological support | 42 (72.4) | 58 |
|  | Motivate relatives for screening | 41 (70.7) |  |
|  | Medical imaging prescription | 36 (62.1) |  |
|  | Clinical examination with breast palpation | 32 (55.2) |  |
|  | I prefer that she be followed by a specialist | 31 (53.4) |  |
|  | Medical advice on choice of care | 30 (51.7) |  |
|  | Care of other pathologies unrelated to *BRCA1/2* | 3 (5.2) |  |
